# Lp-Norm Regularization in Volumetric Imaging of Cardiac Current Sources

**DOI:** 10.1155/2013/276478

**Published:** 2013-11-20

**Authors:** Azar Rahimi, Jingjia Xu, Linwei Wang

**Affiliations:** Rochester Institute of Technology, Rochester, NY 14623, USA

## Abstract

Advances in computer vision have substantially improved our ability to analyze the structure and mechanics of the heart. In comparison, our ability to observe and analyze cardiac electrical activities is much limited. The progress to computationally reconstruct cardiac current sources from noninvasive voltage data sensed on the body surface has been hindered by the ill-posedness and the lack of a unique solution of the reconstruction problem. Common *L*2- and *L*1-norm regularizations tend to produce a solution that is either too diffused or too scattered to reflect the complex spatial structure of current source distribution in the heart. In this work, we propose a general regularization with *Lp*-norm (1 < *p* < 2) constraint to bridge the gap and balance between an overly smeared and overly focal solution in cardiac source reconstruction. In a set of phantom experiments, we demonstrate the superiority of the proposed *Lp*-norm method over its *L*1 and *L*2 counterparts in imaging cardiac current sources with increasing extents. Through computer-simulated and real-data experiments, we further demonstrate the feasibility of the proposed method in imaging the complex structure of excitation wavefront, as well as current sources distributed along the postinfarction scar border. This ability to preserve the spatial structure of source distribution is important for revealing the potential disruption to the normal heart excitation.

## 1. Introduction

Advances in medical imaging modalities have led to an explosion in the quantity and quality of cardiac data available for analysis. Together with the progress in computer vision, there has been a substantial improvement in our ability to assess the structure [1], the kinematics (such as the deformation) [2], and the mechanics (such as the strain distribution) [3] of the heart. Nevertheless, the heart is an electromechanically coupled organ. An efficient contraction of the heart must be preceded by a coordinated electrical current flow throughout the heart muscle. Otherwise a disrupted current flow will directly compromise the ability of the heart to contract and pump effectively. Unfortunately, there is a considerable inadequacy in our ability to observe and analyze the electrical activity and property of the heart.

Electrical currents in the heart, similar to those in the brain, work as bioelectric sources to produce bioelectromagnetic fields that can be sensed as small voltages in the volume conductor of the torso. This voltage change over time is measured on the body surface as electrocardiogram (ECG), similar to the electroencephalogram (EEG) measured for the brain. Biophysical models of this bioelectrical field can be derived from the *quasistatic electromagnetism* [4] where, at any time instant, the *m*-dimensional ECG measurements **b** are described as linear combination of the *n*-dimensional spatial distribution of current source **v**: **b** = **H** **v**. Note that the biophysical model between the current sources in the brain and the EEG signals can be derived from the same physical principle but on a different anatomical region (heart-torso versus brain-skull).

Because there is a lack of experimental techniques to physically measure cardiac electrical signals **v** deep into the thickness of the myocardium, many computational strategies are developed which, analogous to *computed tomography*, aim to computationally *reconstruct* the three-dimensionally distributed, time-varying bioelectrical currents by solving the inverse problem on **b** = **H** **v**, using noninvasive signals **b** collected at different body-surface locations. However, solving this computational inverse problem is afflicted with two sources of challenges. First, this problem is ill-posed and underdetermined because of the limited number of field measurements compared to the large degree of freedom in the unknowns (the possible location of current sources). Errors in the measurement data or the anatomical modeling (reflected in **H**) could highly affect the stability of the solution. Second, even with *virtually* continuous measurements on the surface, this problem still suffers from the lack of a unique solution as determined by the underlying biophysics: different configurations of 3D source distributions may produce the same field measurements on the external surface [4]. Therefore, if the solution is sought transmurally, this inverse problem is intrinsically ill-posed without a unique solution in its most unconstrained form. Proper assumptions of the solutions must be made in order to guarantee a unique solution.

Even though the inverse problem of source reconstruction in the heart (using ECG) and in the brain (using EEG) essentially deals with the same physics problem, developments in the two fields have seen substantial difference in progress. In the latter, numerous approaches have been developed to estimate three-dimensionally distributed current sources [5–7]. In the former, on the contrary, the most commonly used approach is to restrain the solution on the epicardium [8] and/or endocardium [9], sacrificing the information into the depth of the myocardium in exchange for a unique solution. There are few successes in *imaging* the cardiac electrical sources deep into the myocardium, which often involve complex, physiological prior knowledge from computational electrical propagation models of the heart [11, 27]. The question is: *if the two inverse problems are essentially founded on the same physics, what is the obstacle that hinders the progress of cardiac source reconstruction towards a volumetric solution?* We hypothesize that this is, at least in part, caused by the unique spatial property of cardiac current sources. Neural current sources are often focal and compact, and the research focus is to find out which regions in the brain are activated at any given condition. For this purpose, the widely used minimum norm solution (minimum *L*2-norm) [5] was proved to be feasible to provide a solution with minimum overall energy that fits the measured EEG data. Though the solutions are often overly diffused/smoothed, the maximum magnitude in the solution still suffices to approximate the source location. Later, sparse methods such as minimum *L*1-norm [7] and *p*-norm (*p* ≤ 1, realized through recursive weighting scheme) [6] were proposed to obtain sparse solutions that can more accurately pinpoint the location of focal sources.

**Figure 1:**
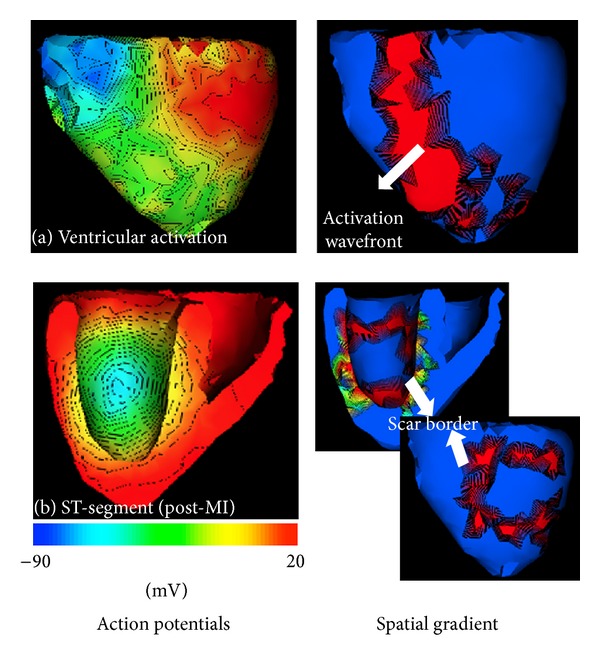
Illustration of the spatial structure of ventricular current sources during a healthy (a) or pathological (b) cardiac cycle.

In comparison, cardiac current source starts from a few focal sites but then propagates throughout the atrial and ventricular myocardium during the cardiac cycle. As a result, the structure of cardiac current sources undergoes a much more complex spatiotemporal change during the cardiac excitation, as illustrated by the two examples given in Figure 1. In a normal depolarization phase of the cardiac excitation, the current sources form an *excitation wavefront* between depolarized and resting cells (Figure 1(a)). After all the cells are depolarized, the heart goes through a stage without current flow (ST-segment in an ECG cycle). Afterwards, the repolarization phase starts and a similar *repolarization wavefront* can be observed to flow throughout the myocardium. In a diseased heart with an infarct, this normal excitation process will be disrupted. For example, during the ST-segment, there will be a voltage difference between healthy myocardium and the center of the infarct, and active current sources will be concentrated along the scar border (Figure 1(b)). This time-varying spatial structure of the current source is important because it reveals the potential disruption to a normal excitation of the heart.

This unique spatial property of cardiac current sources decides that *L*2 or *L*1 regularization will produce a solution that is either too smeared or too focal to reveal the underlying source distribution, even though they have been successful with a similar inverse problem in the brain. Based on this observation, we propose a general regularization with *Lp*-norm (1 < *p* < 2) constraint to cardiac source reconstruction. Balancing between a smeared and a focal solution, *Lp*-norm constraint bridges the gap between *L*1- and *L*2-norm regularizations. The nonlinear *Lp*-norm regularization is solved after being cast to second-order cone programming (SOCP) problem. In a set of phantom experiments, the proposed method is shown to outperform its *L*1 and *L*2 counterparts in imaging cardiac current sources with increasing extents. We further demonstrate the feasibility of the proposed method in imaging the complex structure of excitation wavefront during a normal propagation (Figure 1(a)), as well as that of the scar border during a ST-segment in an infarcted heart (Figure 1(b)). An initial real-data experiment also attests to its feasibility in detecting scar border in a postinfarction human subject.

## 2. Related Works

As mentioned earlier, the quasi-static electromagnetism governs the relation between cardiac current sources and body-surface voltage measurements. Employing proper numerical methods such as mesh-free and boundary element methods, this relationship can be rendered to **b** = **H** **v**, where **H** is built on a subject-specific heart-torso model in ECG source localization problem. To overcome the ill-posedness of the inverse problems on these bioelectric fields, proper regularization needs to be employed:

(1) $$\begin{array}{c} \underset{v}{\min}\;{||b-Hv||}_{2}^{2}+\lambda C\left(v\right), \end{array}$$

where the first term in the objective function describes the least square minimization of data-fitting error (data fidelity term) and the second term defines the regularization constraint. *λ* is the regularization parameter that controls the trade-off between fitting to the data and comforting to the constraint.

### 2.1. *L*2 Regularization: Smooth Constraints

The most common constraint used in (1) is *L*2 or weighted-*L*2 norm to enforce the smoothness of the source distribution: *C*(**v**) = ||**F** **v**||_2_^2^, where **F** is usually defined as identity matrix, gradient operator, and Laplace operator for 0-order, 1-order, and 2-order regularization, respectively. In *L*2-norm regularization, regularization parameter *λ* is typically determined using the generalized cross-validation, the discrepancy principle, and the *L*-curve method [12].

The mainstream approaches, addressing the inverse problem of ECG source localization on the heart surfaces, are mainly based on *L*2-norm regularization using different spatial and/or temporal constraints. These methods include Tikhonov regularization method [8], least squares QR (LSQR) [13], truncated total least square (TTLS) [14], Kalman filter [15], generalized minimal residual [16], and level-set [17] and statistical approaches [18]. Although incorporating the *L*2-norm-based constraint handles the ill-posedness of this inverse problem and provides stability in the presence of noise, it ultimately diffuses the source reconstruction solution. The smoothing nature of *L*2 regularization makes it infeasible to trace the complex spatial distribution of the cardiac current sources using the region with maximum energy (as shown in Section 4.2).

*L*2-norm-based regularization was later extended to a 3D setting in order to *image* volumetric current sources in the heart [19, 20]. Employing a weighted *L*2-norm regularization on the intramural solution, these methods successfully estimate the active sources during the initiation sites and activation sequence [19] and the ST segment of an ECG cycle [20]. Because of the simplicity of the constraint, these methods can only be utilized to recover the source activity during one stable period of cardiac excitation cycle where the source distribution does not go through notable temporal changes. To consider the complete temporal changes of the cardiac sources in a complete excitation cycle, more complex prior knowledge in terms of 3D intramural electrical excitation model of the heart was included in [11, 27]. *L*2 penalty is then used to enforce the solution to be close to that predicted by the computer model. While being able to reconstruct the complete spatiotemporal changes of the current sources, this type of approaches is influenced by the prior knowledge produced by the excitation model; furthermore, due to the *L*2-norm penalty, the solution only renders patternwise qualitative accuracy but loses quantitative accuracy in the distribution of 3D current sources [21].

### 2.2. *L*1 Regularization: Sparse Methods

The most popular approach to circumvent the smoothing effect of *L*2-norm constraint is to employ *L*1-norm penalty during regularization [22] *C*(**v**) = ||**F** **v**||_1_, where **F** is defined similar to that in the *L*2-norm constraint. For this type of approaches, there is no established methods to objectively set the value of the regularization parameter *λ*, and common practice resorts to empirical methods depending on the dataset.

As explained earlier (Figure 1), cardiac current sources are often localized, but not focal, during the course of a cardiac cycle. Therefore, sparse methods are rarely considered in the ECG inverse problem. Recently, *L*1 regularization was introduced for the first time to improve the sharp features of the source reconstruction on the epicardium [23, 24]. While it has been shown to numerically improve the resolution of the solution, it is unknown if the sparsity assumption is tied to the physiological property of the epicardial equivalent source models. In another work, *L*1-norm was extended to the data term in order to improve the solution in terms of outliers [25]. Most recently, we developed and demonstrated the efficacy of a *Lp* regularization (*p* ≤ 1) based on recursive weighting scheme to successfully pinpoint the focal sources in the beginning of an electrical propagation cycle [26]. However, as explained earlier, such focal sparsity of cardiac current sources is quickly lost as the current flows throughout the heart muscle, and the same sparse method is no longer applied. The regularization method that imposes sparsity at the early stage of electrical excitation, therefore, must be able to adapt to this change of spatial property of the current sources for the rest of the cardiac cycle.

## 3. Methodology

Based on the quasi-static electromagnetism [4], the potential distribution within torso volume conductor is produced by the cardiac current sources according to

(2) $$\begin{array}{c} \sigma_{\text{blk}}\nabla^{2}\phi_{e}\left(r\right)=\nabla \cdot \left(-D_{\mathrm{int}}v\left(r\right)\right),\;\forall r\in \Omega_{h}, \end{array}$$

(3) $$\begin{array}{c} \sigma \nabla^{2}\phi \left(r\right)=0,\;\forall r\in \Omega_{t/h}, \end{array}$$

where the Poisson equation (2) describes, on a bidomain heart model, how the extracellular potential *ϕ*_*e*_ within the heart volume *Ω*_*h*_ originates from the current sources *v* modulated by the anisotropic intracellular conductivity tensor **D**_int⁡_. *σ*_blk_ is the bulk conductivity assumed to be isotropic. The Laplace equation (3) describes, on the monodomain torso model, how the potential *ϕ* distributes within the torso volume *Ω*_*t*/*h*_ external to the heart with conductivity *σ*.

**Figure 2:**
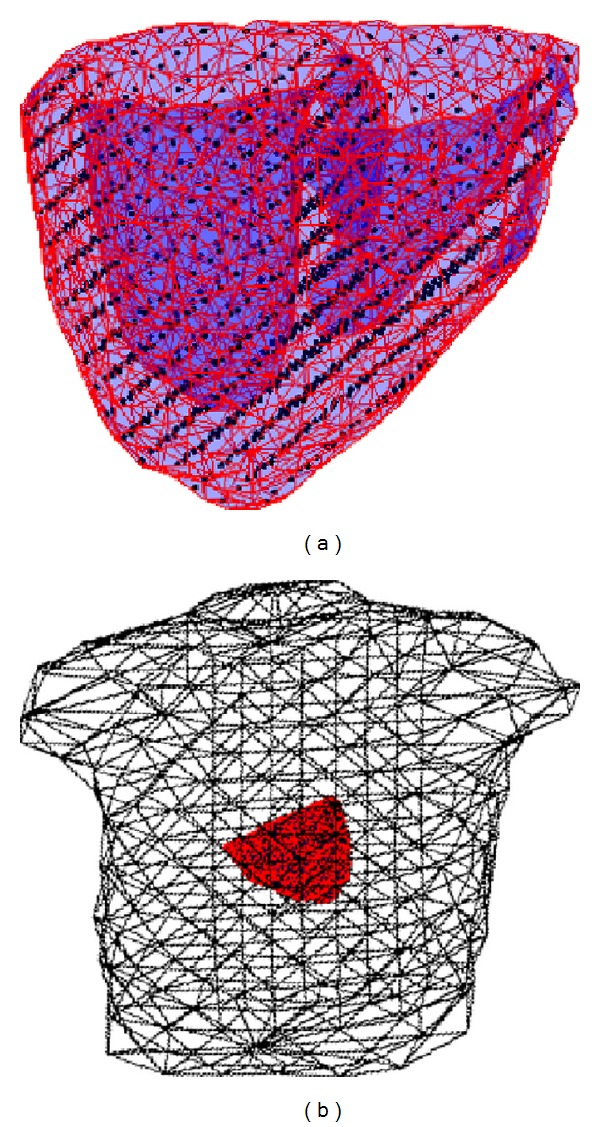
(a) Mesh-free nodes (black points) that represent the 3D myocardium. (b) Coupled heart-torso model.

We have previously shown that, using mesh-free and boundary element methods, we can numerically solve (2) and (3) on a given heart-torso model of a subject (Figure 2) and obtain a linear relationship between ECG measurements (**b**) and the current sources (**v**): **b** = **H** **v** [10].

### 3.1. *Lp*-Norm Regularization

As mentioned earlier, reconstructing 3D current sources from ECG data is a highly ill-posed inverse problem with nonunique solutions in its most unconstrained form. However, complex spatial distribution of cardiac current sources conflicts with a focal *L*1 or smooth *L*2 constraint. To estimate the special structure of current sources, we apply *Lp*-norm regularization:

(4) $$\begin{array}{c} \underset{v}{\min}\;{||b-Hv||}_{2}^{2}+\lambda {||v||}_{p},\;1<p<2,\\ {||v||}_{p}={\left(\sum_{i=1}^{n}{\left[v_{i}\right]}^{p}\right)}^{1/p}, \end{array}$$

where *n* is the dimension of **v**, that is, the number of mesh-free nodes used to represent the ventricular myocardium.

*Lp*-norm penalty term promotes different forms of structural sparsity as often observed in the heart. It offers the potential to outperform sparse *L*1-norm and diffused *L*2-norm for localizing sources with different extents/sizes.

### 3.2. *p*-Order Cones and Second-Order Cones Programming

Solving this *Lp*-norm regularization is not possible using linear or quadratic programming. Here we adopt SOCP that allows minimization of linear objective functions with quadratic cone constraints based on interior point methods. Furthermore, it provides flexibility to incorporate an arbitrary number of constraints while providing an efficient solution. To do so, we need to first reformulate our inverse problem (4) into a *p*-order cone programming (*p-OCP*) problem, which can be obtained by introducing two intermediate variables *ξ*, *η* into the objective function:

(5) min⁡ξ,η  ξ+ληs.t.  ||b−Hv||22⩽ξ||v||p⩽η.

Assuming *p* as a positive rational number (*p* = *r*/*s*), this *p-OCP* problem can then be transformed into a set of linear inequalities and 3D SOC constraints and be handled by SOCP methods [28]. In this way, the conic constraint (*v*_1_^*p*^ + ⋯+*v*_*n*_^*p*^)^1/*p*^ ⩽ *η* is equivalent to

(6) $$\begin{array}{c} v_{j}^{r}⩽u_{j}^{s}t^{r-s},\;u_{j}⩾0,\;j=1,\ldots ,n\\ t⩾\sum_{j=1}^{n}u_{j}. \end{array}$$

Each constraint is then represented by a sequence of 3D rotated SOC constraints that can be expressed with inequalities of the form *z* ^2^ ⩽ *xy*.

## 4. Results

### 4.1. Imaging Current Sources with Various Extents

First, we consider synthetic experiments on a heart-torso model derived from a human subject as shown in Figure 2. The torso surface is represented by triangulated elements with 370 vertices. The ventricular myocardium is represented by total 1019 nodes distributed in a cubic grid with 7 mm intergrid distance and confined by the ventricular surface.

In the first set of experiments, we investigate the performance of *Lp*-norm regularization in localizing current sources with different sizes. In total, 44 settings are studied, considering a region of active current sources sized from 1% to 45% of the left ventricle. These sources form a region with structural sparsity located randomly inside the ventricular myocardium. The nodes lying within the region of active sources are assigned with values 1, while the rest of the ventricular nodes are set to be 0. For each setting, the corresponding ECG measurements are simulated on the 370 vertices on the body surface and are corrupted with white Gaussian noise before being input to the *Lp*-norm method to reconstruct the region of active current sources. The accuracy of 3D source estimation is evaluated using the *source overlap* (SO) defined as the intersection divided by the union between the estimated and the *true* region of current sources:

(7) $$\begin{array}{c} \text{SO}=\frac{\text{simulated}\;\text{sources}\cap \text{estimated}\;\text{sources}}{\text{simulated}\;\text{sources}\cup \text{estimated}\;\text{sources}}. \end{array}$$

Setting *p* value to 1 and 2, we also perform *L*1- and *L*2-norm estimation of the 3D current source distribution and compare the results with the proposed *Lp*-norm method.

#### 4.1.1. Values of *p* versus Source Extents

For every source setting, *Lp*-norm estimation is obtained using *p* ∈ {1.1,1.3,1.5,1.7,1.9} and is compared to that obtained by *L*1 and *L*2 solutions. 30 dB noise is considered.

**Figure 3:**
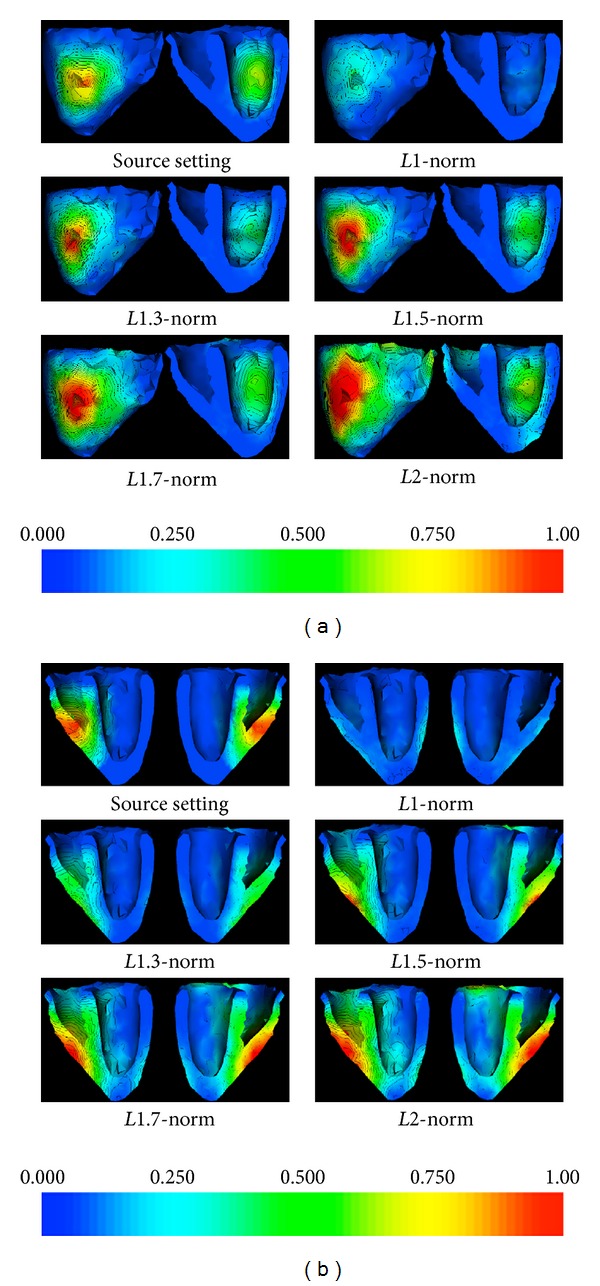
Source estimation using *Lp*-norm regularization for 1 ≤ *p* ≤ 2. (a) 58 active sources are located at left ventricle mid-anterior. (b) 127 active sources are located close to the right ventricle apex. Increasing the *p* value increases the source extent such that *L*1-norm obtains too scattered source distribution while *L*2-norm provides overly diffused solution. Current magnitudes are normalized to 1.

Figure 3(a) shows an example of source estimation using *Lp*-norm regularization for 1 ≤ *p* ≤ 2, where the active region is located at mid-inferior of the left ventricle. The *L*1-norm estimation of active sources results in a very sparse source reconstruction (SO = 0.05) scattered in the *true* region of active sources, and nearly no active sources were detected close to the endocardium. Increasing the *p* value for the *Lp*-norm regularization, the detected source extent increases. At *p* = 1.3, we obtain an accurate estimation of source extent (SO = 0.38), which is located very close to the *true* region of active sources. As *p* continues to increase, the estimated source region becomes more extended but still has a relatively compact center. There is a sudden change of pattern in the solution when *p* equals 2, where the estimated source region (*L*2 solution) becomes very diffused (SO = 0.22). Another example is presented in Figure 3(b), where the active current sources are located close to the right ventricle apex. Similar pattern can be observed in the source estimation by increasing the *p* value from 1 to 2.

**Figure 4:**
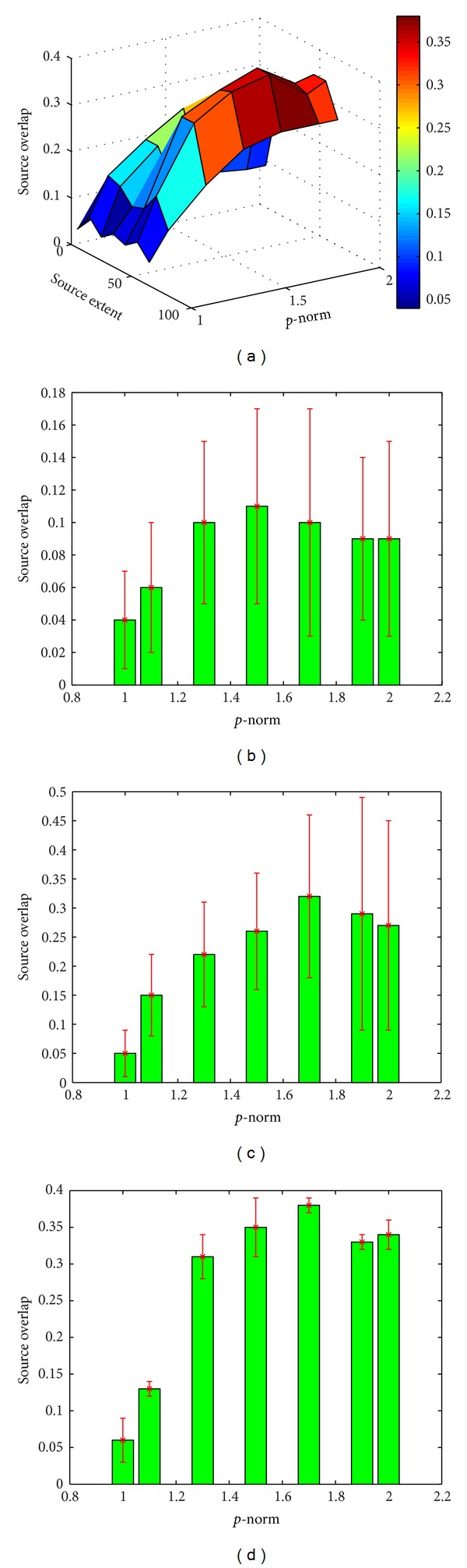
(a) Source overlap (SO, vertical axis) obtained by *Lp*-norm reconstruction (1 ≤ *p* ≤ 2, horizontal axis 1) for active sources with different extents (ranging from 1 to more than 80 active sources in the region, horizontal axis 2). (b)–(d) Examples of SO mean and standard deviation obtained by *Lp*-norm reconstruction (1 ≤ *p* ≤ 2) for a region of (b) 1–10, (c) 20–30, and (d) 40–50 active sources.

Figure 4(a) summarizes the mean SO (vertical axis) between the *true* and estimated source regions obtained using *Lp*-norm regularization, as *p* increases from 1 to 2 (horizontal axis 1) and as the size of active region increases (horizontal axis 2). As shown, for source regions of all sizes, similar trend of SO change can be observed as *p* increases from 1 to 2 in the *Lp*-norm regularization: the sparse solution produced by *L*1-norm regularization, though produces low false-positives, also has a high underestimation (low numerator in the calculation of the OS) and therefore a low value of OS. On the other extreme, the smeared solution of *L*2-norm regularization, though is able to detect the majority of the *true* active sources, tends to have a high overestimation (high denominator in the calculation of the OS) and thus leads to again a low OS value. Therefore, for source region of all sizes (as the 3 examples shown in Figures 4(b)–4(d)), we can observe an increase followed by a decrease of the OS value when *p* increases from 1 to 2, with the maximum OS obtained when 1.5 ≤ *p* ≤ 1.6. Such benefits of the *Lp*-norm regularization with 1 < *p* < 2 are particularly evident when the source region is of medium size (≤30% of the left ventricle).

#### 4.1.2. Noise Effect on *Lp*-Norm Source Estimation

**Figure 5:**
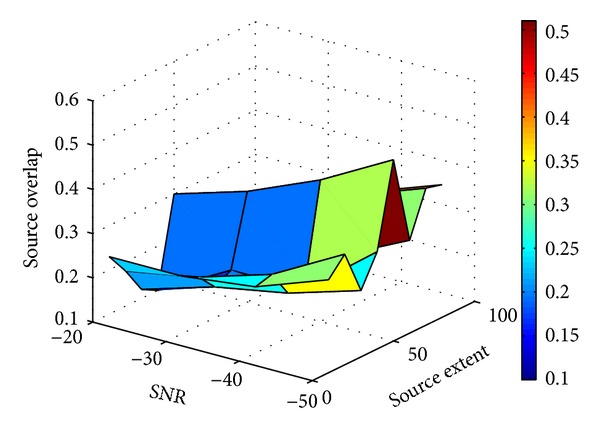
Source overlap (SO, vertical axis) obtained by *Lp*-norm reconstruction (*p* = 1.5) for active sources with different extents (horizontal axis 1) in presence of white Gaussian noise with different SNR levels (horizontal axis 2).

Next, we investigate the performance of our proposed *Lp*-norm regularization in presence of noise with different SNR levels (50–20 dB), using *p* = 1.5 as an example. Here we consider a region of size 1–52% of the left ventricle. As shown in Figure 5, increasing the noise level leads to minor decreasing of the OS value, and the trend of change is similar for sources of all sizes. The mean SO calculated for different source extents in presence of 50 dB noise is 0.35 and starts to decrease to 0.28, 0.25, and 0.2 as the SNR decreases to 40, 30, and 20, respectively. Again, the advantage of *Lp* regularization is more evident when the source is of medium size (~30% of LV).

### 4.2. Computer-Simulated Electrical Activity

As explained earlier, one critical feature of cardiac current sources that differs from neural current sources is the complex spatial structure they exhibit during the cardiac cycle of electrical propagation, which is likely to be the cause of the difficulty of using *L*1 or *L*2 regularization for faithful reconstruction. In this set of experiments, we increase the complexity of the experimental settings and consider *realistic* structures of current sources, which are generated by computer simulations of the spatiotemporal propagation of electrical waves in the ventricles.

#### 4.2.1. Imaging Excitation Wavefront

**Figure 6:**
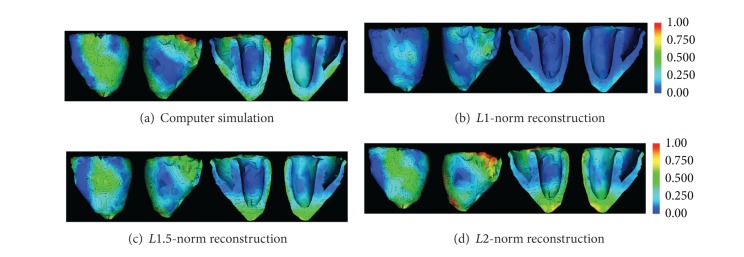
Excitation wavefront estimation using *Lp*-norm regularization versus *L*1- and *L*2-norm counterparts.

First, we consider the ability of the proposed *Lp*-norm regularization in reconstructing the complex structure of excitation wavefront.  Figure 6(a) shows an example of an excitation wavefront during a normal propagation in a healthy ventricle at 33 ms after the onset of ventricular excitation. Similar to our earlier observations, the *L*1 reconstruction produces scattered solution where the spatial structure of the excitation wavefront is lost (Figure 6(b)). The *L*2 reconstruction, on the other extreme, produces a blurred region of activation where the structure of excitation wavefront is smeared (Figure 6(d)). In comparison, the *Lp* reconstruction (*p* = 1.5) better preserves the excitation wavefront (Figure 6(c)). Quantitatively, the *Lp* regularization obtains OS = 0.26, while the *L*1 solution provides OS = 0.07, and the *L*2 solution produces OS = 0.23.

The ability to properly capture the spatial structure of the excitation wavefront, with a solution neither too scattered nor too diffused, is important because tracing the excitation wavefront can reveal the existence and location of obstacles that disrupt the normal propagation of electrical waves.

#### 4.2.2. Imaging Source Localization along the Scar Border

Second, we examine the feasibility of the proposed method in estimating the current source activity along the scar border in an infarcted heart. As explained earlier, during the ST-segment of an ECG cycle, there is no current flow in a healthy heart. In an infarcted heart, in comparison, only the viable myocardium would exhibit coherent high voltage, while the necrotic tissue in the scar core will exhibit low voltage. These two regions will be separated by the scar border where the active current sources are localized. 

**Figure 7:**
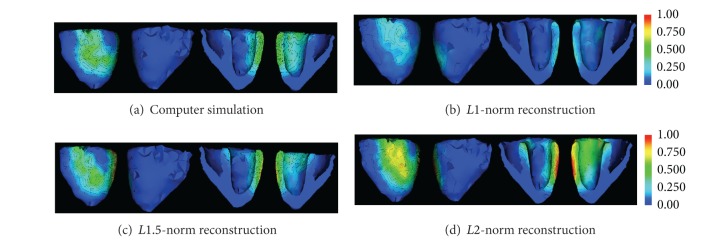
Estimation of current sources localized along the scar border using *Lp*-norm regularization versus *L*1- and *L*2-norm counterparts.

Figure 7 shows an example of current source distributed along the border of an infarct that extends from basal to mid-anterior and anterolateral LV. *Lp* regularization (*p* = 1.5) detects an active sources region consistent with the *true* scar border, reporting an SO = 0.35. In comparison, the *L*1 regularization produces a scattered solution (SO = 0.08) and the *L*2 regularization produces a diffused solution (SO = 0.26), neither of which is able to capture the structure topology of the current sources along the scar border. This ability of the proposed *Lp* reconstruction to faithfully reconstruct the current sources distributed along the scar border is of great importance because the scar border is known as the common site for triggered electrical activity and reentrant circuits that can initiate and maintain life-threatening ventricular arrhythmias.

### 4.3. Real-Data Experiments: Imaging Scar Border for a Postinfarction Patient

Because of the important therapeutic value of scar border, and the promising results obtained from our initial synthetic experiments, we continue to conduct an initial real-data experiment on a real post-infarction human subject to assess the feasibility of the proposed *Lp*-norm method in detecting current sources along the scar border.

**Figure 8:**
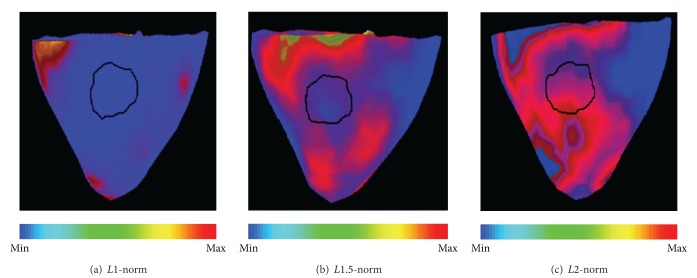
Estimation of current sources localized along the scar border using *Lp*, *L*1- and *L*2-norm regularizations for a post-infarction human subject.

Experimental data were collected from a patient with prior myocardial infarction and made available to this study by *2007 PhysioNet/Computers in Cardiology Challenges* [29]. MRI scan of the patient has 8 mm interslice spacing and 1.33 mm/pixel in-plane resolution. Body surface ECG maps were recorded by *Dalhousie University* standards [30] at 123 known anatomical sites and interpolated to 370 nodes of the *Dalhousie* torso model; each BSP recording consists of a single averaged PQRST complex sampled at 2 kHz. Gold standards of the scar were provided by cardiologists who examined the late Gadolinium enhanced (LGE) MR scans of the patient, and were provided in terms of the location and size of the scar with regard to the 17-segment division of the LV according to AHA standards [31]. Specifically, according to the gold standard, the scar center is located at segments 10 and 11, between mid-inferior and mid-inferolateral of the subject's left ventricle (highlighted with black contour in Figure 8).

ECG data collected at the 192 ms during the ST-segment are selected as the input data. As shown in Figure 8(a), the *L*1 regularization results in a very sparse solution scattered far from the infarct center. Regions of current sources provided by *L*2 regularization (Figure 8(c)) are diffused and cover the scar center; that is, the structure of the scar border cannot be discerned by the reconstruction. The proposed *Lp* solution (*p* = 1.5) (Figure 8(b)) provides a more accurate estimation of the current sources, which can be seen to distribute around the center of the scar.

## 5. Discussions and Conclusions

The inverse problem of cardiac source reconstruction is notoriously ill-posed without a unique solution. Progress towards volumetric cardiac source reconstruction is further hindered by the complex structure of current source distribution in the heart because of which the common *L*1- and *L*2-norm constraints are no longer proper because they make an assumption that is either too focal or too smooth regarding the source distribution.

Our experiments' results on localization of current source activity (Section 4.1.1) show that *L*1-norm constraint only works well in recovering focal sources. As a result, it can be employed in the applications where the target source is sparse and focal such as detecting the pacing sites as described in [23–25]. Increasing the size of active source region decreases the performance of *L*1-norm such that the detected sources are too sparse to provide any information about the structure of source region. These extended source regions occur, for example, as activation and repolarization wavefronts during depolarization and repolarization stages of a cardiac cycle, as shown in Figure 1. In comparison, *L*2-norm regularization provides better approximation of extended source regions while it provides an overly smeared estimation of focal and compact source regions. Because of *L*2-norm smoothing effect, it fails to distinguish multiple proximal active current sources.

We proposed a general *Lp*-norm regularization to bridge the gap between the scattered and smeared solutions of *L*1 and *L*2 regularizations and show its potential in imaging cardiac current source distributions that are of important therapeutic information, such as the excitation wavefront, and the source distribution along the myocardial scar border in an infarcted heart. *Lp*-norm provides a solution that better reflects different spatial properties of cardiac current sources. Our results show its better performance in detecting current sources with different extents compared to *L*1- and *L*2-norms.

It should be noted that our work focuses on estimation of volumetric current sources whose spatial and temporal properties are different from those of an equivalent source model such as the potential distribution on the epicardium [23–25]; while the spatiotemporal dynamics and properties of volumetric current sources (true cardiac sources) are well known and can be well deduced from our knowledge of the physiology of cardiac excitation [32], the spatiotemporal physiological property of epicardial potential as an equivalent source model is not clear. Therefore, the conclusion drawn for one source model regarding which types of regularization would achieve the best performance cannot be directly extended to the other. In addition, in this work the accuracy of source localization is presented in terms of source overlap compared to other surface-based approaches that use relative error and correlation coefficient as accuracy measures [23–25].

At the current stage, the *Lp*-norm reconstruction is separately performed at each time instant of the measured ECG data. Therefore, temporal information of the electrical current flow in the heart is not taken into account. Because the current flow follows a diffusion process, temporal relation between consecutive time instants has the potential to improve the stability and accuracy of the reconstruction and will be studied in the next step of this research.

It is also observed that, when *p* increases from 1 to 2, the accuracy of source reconstruction increases then decreases. Our experimental results show that the optimal solutions are obtained at *p* = 1.5–1.6 for sources with different sizes, though a larger *p* is often needed as the size of the source increases (Figure 4). In this feasibility study, *p* = 1.5 is considered for the computer-simulated and real-data experiments. Because it is not possible to foresee the size of the source before the reconstruction, in the future we will investigate the possibility to simultaneously estimate the value of *p* during the *Lp*-norm regularization, where the optimal value of *p* can be decided by the datasets under study.
